# Shared *T*-Cell Receptor Repertoire in the Tonsils of Patients with IgA Nephropathy

**DOI:** 10.34067/KID.0000001154

**Published:** 2026-02-10

**Authors:** Kazunori Satokata, Shin Goto, Hiroki Yamaguchi, Hirofumi Watanabe, Nao Takahashi, Koichi Higashi, Suguru Yamamoto, Yoshikatsu Kaneko, Arata Horii, Ken Kurokawa, Ichiei Narita

**Affiliations:** 1 Division of Clinical Nephrology and Rheumatology, Kidney Research Center, Niigata University Graduate School of Medical and Dental Sciences, Niigata, Japan; 2 Department of Otolaryngology Head and Neck Surgery, Niigata University Graduate School of Medical and Dental Sciences, Niigata, Japan; 3 Genome Evolution Laboratory, National Institute of Genetics, Mishima, Japan

**Keywords:** gene expression, glomerular disease, GN, IgA nephropathy, immunology

## Abstract

**Key Points:**

- Patients with IgA nephropathy have significantly more abundant and shared joining regions in TCR*α* repertoires compared with those with recurrent tonsillitis.
- These are characterized by shorter complementary determining region 3 lengths and low mucosal-associated invariant *T* match scores.
- The clonotypes are correlated with the IgA-binding index of bacteroidetes and with tonsillar galactose-deficient IgA1 in these patients.

**Background:**

Aberrant mucosal immune responses are underlying causes of IgA nephropathy (IgAN), the most prevalent type of chronic GN. However, the role of *T* cells in IgA nephropathy pathogenesis remains elusive. To address this knowledge gap, we profiled the *T*-cell receptor repertoire in the tonsils of patients with IgA nephropathy.

**Methods:**

This study included 27 and 20 patients with biopsy-confirmed IgA nephropathy and recurrent tonsillitis (RT), respectively, who underwent tonsillectomy. The *T*-cell receptor (TCR) repertoire was determined by high-throughput sequencing coupled with unbiased adaptor ligation PCR. Furthermore, the usage of variable and joining regions in TCR*α* (TRA) and joining regions in TCR*β* (TRB) genes in each group was assessed. TRA clonotypes shared among the patients were characterized by complementary determining region 3 lengths, types of mucosal-associated invariant *T* (MAIT) cells, hydrophobicity, and their relationships with tonsillar galactose-deficient IgA1 and tonsillar IgA-binding indices of tonsillar bacteria.

**Results:**

The TRA repertoire exhibited significantly lower similarity in patients with IgA nephropathy than did in RT cases (*P* < 0.001). Sharing TRA clonotypes among patients with IgA nephropathy was significantly sparser than that among RT cases. The relative abundance of shared TRA clonotypes with shorter complementary determining region 3 lengths was significantly increased in patients with IgA nephropathy (*P_adj* = 0.041), which was characterized by low MAIT match scores. Significant negative correlations were observed between the MAIT scores and hydrophobicity for these TRA clonotypes in patients with IgA nephropathy. The relative abundances of these clonotypes significantly and positively correlated with the IgA binding indices of the phylum Bacteroidetes (*P_adj* = 0.008) in both groups and tonsillar galactose-deficient IgA1 levels in patients with IgA nephropathy (*P* = 0.035).

**Conclusions:**

The results in this study suggest aberrant *T*-cell subsets involvement in tonsillar immunity in patients with IgA nephropathy.

## Visual Abstract

**Figure:**
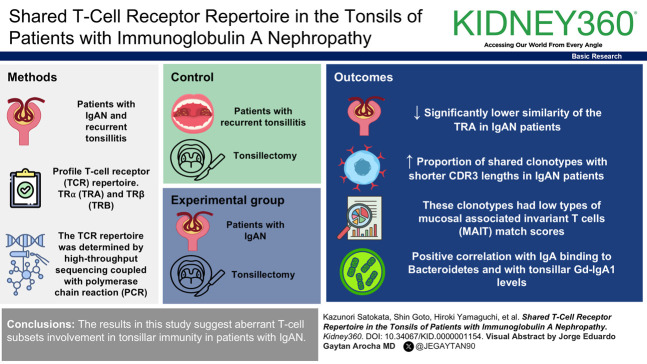

## Introduction

IgA nephropathy is pathologically characterized by poor O-galactosylated IgA1 deposits in the mesangium.^1^ Patients with IgA nephropathy often express exacerbated urinary symptoms triggered by tonsillitis or upper respiratory tract inflammation, suggesting mucosal immune response involvement—including that of the tonsils—in IgA nephropathy pathogenesis. Data from Japan indicate that tonsillectomy, in addition to immunosuppressive therapy, is effective against IgA nephropathy.^2^

Aberrant immune responses triggered by immune tolerance breakdown in resident bacteria have been associated with O-galactosylated IgA1 production.^3^ *T*-cell–dependent and independent pathways have been shown to influence IgA class switching in B cells, generating high-affinity and low-affinity IgA antibodies, respectively. Regarding *T*-cell–dependent IgA class-switching, *T* cells specifically signal B cells through the CD40 ligand and TGF-*β*1, whereas *T*-cell–independent IgA class switching requires innate signals derived from microbial Toll-like receptor ligands and CD40 ligand-related mediators, such as the B-cell–activating factor and proliferation-inducing ligand (APRIL).^4^ Regarding tonsillar crypts, we recently reported significantly elevated expression of proliferation-inducing ligand and B-cell–activating factor in patients with IgA nephropathy compared with patients with recurrent tonsillitis (RT). Correspondingly, a specific repertoire of IgA heavy chains is preferentially expressed, suggesting that *T*-cell–independent pathways are aberrantly involved in the mucosal immune response in patients with IgA nephropathy.^5^

Other studies have shown that innate and adaptive immunity involving *T* cells may play critical roles in IgA nephropathy pathogenesis.^6-9^ Although data suggest the potential involvement of tonsillar *T* cells in IgA nephropathy pathogenesis, prior assessments have been limited and it remains unclear whether innate-like or adaptive *T*-cell immune responses are primarily responsible for the pathogenesis of IgA nephropathy. Dissecting these responses requires comprehensive profiling to identify distinct *T*-cell populations and define their phenotypic characteristics.

Advances in high-throughput immune repertoire sequencing technologies have enabled analysis of a large number of immune cells in biologic samples.^10^ Investigating the diversity of *T* cells by sequencing the repertoire of *T*-cell receptors (TCR) and identifying the specific repertoire of *T* cells is currently in progress.^11,12^ Rosati *et al*. identified a semi-invariant TCR*α* (TRA) chain abundant in patients with Crohn's disease and called the immune cells harboring it as unconventional Crohn's disease–associated invariant *T* cells.^13^ Linsley *et al*. analyzed the public TCR*α* chain shared between islet antigen-reactive and peripheral blood *T* cells and determined its influence on type 1 diabetes onset.^14,15^ These examples in immune-mediated diseases highlight the considerable potential of TCR repertoire analysis to identify disease-specific *T*-cell populations.

Considering the aberrant innate immunity in patients with IgA nephropathy, investigating the alterations in these innate-like TCRs is crucial to understand the basis of immune abnormalities in patients with IgA nephropathy. In this article, we analyzed the repertoire of *T*-cell receptors in tonsillar tissues and demonstrated the involvement of a distinct population of TCR repertoire subsets in abnormal mucosal immune responses in the tonsils of patients with IgA nephropathy.

## Methods

### Enrolled Patients and Collected Specimens

The Ethics Committee on Genetic Analysis of Niigata University approved this study (approval no. G2017-0004, G2022-0010); all enrolled individuals provided written informed consent. Tonsillar samples were obtained from patients with IgA nephropathy and RT who underwent tonsillectomies between July 2012 and June 2018, as previously described.^16^ We excluded one sample (IgAN16) since it exhibited reduced diversity in both joining regions in TRA and joining regions in TCR*β* (TRB), making it an outlier.

### Unbiased Amplification of TCR Genes and Deep Sequencing of TCR Repertoire

**Figure 1:**
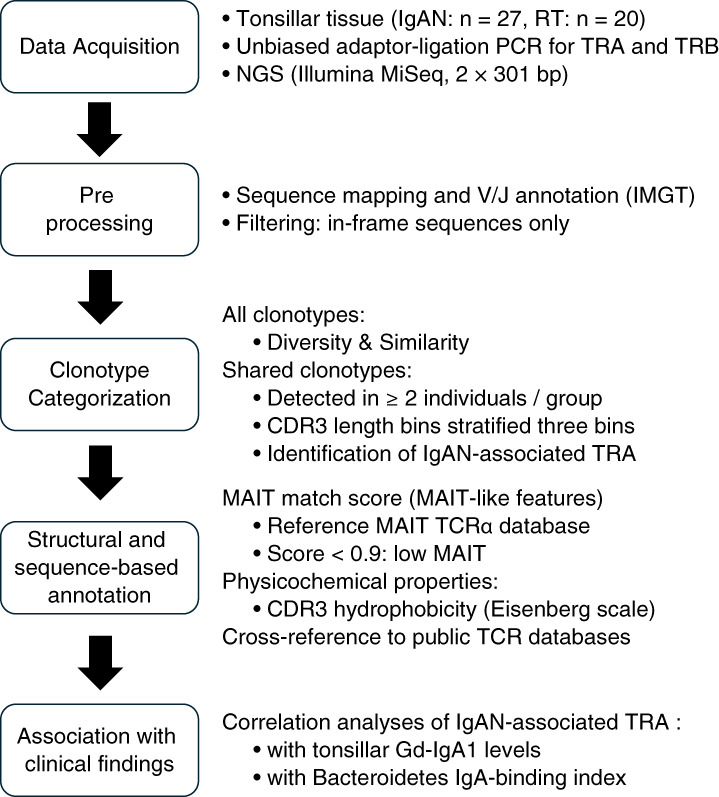
**Pipeline of TCR repertoire analysis using tonsillar tissue.** This figure shows a schematic overview of the TCR repertoire analysis workflow using tonsillar tissue. The right panel depicts the analytical process, including data acquisition, preprocessing, clonotype categorization, structural and sequence-based analyses, and association analyses with clinical findings. A brief summary of each step is provided in the right panel. CDR3, complementary determining region 3; Gd-IgA1, galactose-deficient IgA1; IMGT, the International ImMunoGeneTics Information System; MAIT, mucosal-associated invariant *T*; RT, recurrent tonsillitis; TCR, *T*-cell receptor; TRA, joining regions in TCR*α*; TRB, joining regions in TCR*β*.

A schematic overview of the TCR repertoire analysis workflow is shown in Figure 1. We extracted total RNA from approximately 30 mg of tonsillar tissue, and unbiased adaptor-ligation PCR and TCR sequencing were conducted as previously reported (Supplemental Table 1).^17^ In brief, we used the ds-cDNA synthesized from 2 *µ*g of total RNA, ligated it with a P10EA/P20EA adaptor, and digested it with NotI (TaKaRa, Shiga, Japan). With primers targeting the P20EA and C-regions of TRA and TRB (CA1 and CA2 and CB1 and CB2, respectively), nested PCR was performed using the following protocol: 20 cycles of 95°C for 20 s, 60°C for 30 s, and 72°C for 1 minute. Then, to prepare PCR amplicons, the nested PCR products were amplified using P22EA-ST1-R and C region–specific Tag primers (CA-ST1-R and CB-ST1-R) and purified using AMPure XP magnetic purification beads (Beckman Coulter, Brea, CA). Finally, index sequences were added to the PCR amplicons using the Nextera XT Index Kit v2 (Illumina, San Diego, CA). Equal amounts were subjected to paired-end sequence (2×301 base-pair) on the Illumina MiSeq platform. After filtering for quality control, 7,213,585 and 6,827,961 sequence reads were obtained from the tonsil samples of 27 patients with IgA nephropathy and 20 with RT, respectively. A total of 2,321,020 and 1,614,568 sequence reads in patients with IgA nephropathy were assigned for TRA and TRB repertoire analysis (mean reads±SD, TRA: 85,963±37,267, TRB: 59,798±21,612), respectively, and 1,643,365 and 1,267,671 sequence reads in patients with RT were assigned for TRA and TRB repertoire analysis (mean reads±SD, TRA: 82,168±28,003, TRB: 63,383±21,632), respectively. The remaining 2,085,168 and 1,507,110 in-frame sequence reads in patients with IgA nephropathy (mean reads±SD, TRA: 77,228±33,238, TRB: 55,818±20,361) and 1,483,042 and 1,181,058 in-frame sequence reads in patients with RT (mean reads±SD, TRA: 74,152±24,957, TRB: 59,052±20,372) were used for TRA and TRB repertoire analysis, respectively (Supplemental Table 2). We determined the clonotypes of TRA and TRB using in-frame sequence reads from each patient in both groups (Supplemental Table 3).

### TCR Repertoire Analysis Using Bioinformatics Tools

The usage of variable and joining regions and in TRA and TRB genes of tonsils was characterized, and their clonality and diversity were assessed using bioinformatics software (Repertoire Genesis Incorporation, Ibaraki, Japan).^18,19^ We compared the reads with the reference sequence of *T* cell receptor alpha variable (TRAV), *T* cell receptor alpha joining (TRAJ), *T* cell receptor beta variable (TRBV), and *T* cell receptor beta joining from the International ImMunoGeneTics Information System database website (http://www.imgt.org/). V and J regions were annotated automatically through Repertoire Genesis, an original in-house program. To evaluate similarities of TCR repertoires at the clonal level between individuals divided into IgA nephropathy and RT, we retrieved the number of TCR sequence clonotypes shared among more than two samples in each group and calculated the Morisita-Horn index derived through the mh function in the divo package of the *R* program.^20^ We determined the ratio of the number of shared clonotypes in each sample to the total number of shared clonotypes observed. For this analysis, shared clonotypes were defined as those with more than two reads and shared among more than two patients within the same group (IgA nephropathy or RT). Clonotypes not meeting this criterion were considered unshared or private. In-frame reads not identified as a single clone in each gene segment were removed before the analysis. To estimate the characteristics of shared TRA clonotypes, we calculated the mucosal-associated invariant *T* (MAIT) match scores using similarity in complementary determining region 3 (CDR3) sequences between the shared TRA clonotypes and a reference database of MAIT sequences (http://www.cbs.dtu.dk/services/MAIT_Match). TRAV and TRAJ gene usage in the clonotype of IgA nephropathy-associated TRAs was depicted using ggalluvial: Alluvial Plots in ggplot2. *R* package version 0.12.5 (http://corybrunson.github.io/ggalluvial/).^2.1^

The hydrophobicity of the amino acids in the CDR3 region of the TRA clonotype was calculated as the average hydrophobicity of each amino acid, as defined by the Eisenberg scale.^22^

The clonotype of IgA nephropathy-associated TRAs was searched using databases such as the a curated database of T-cell receptor sequences with known antigen specificities (VDJdb),^23^ Unconventional *T*-cell receptor database (UcTCRdb),^24^ and pathology-associated TCR database.^25^

### Statistical Analysis

Data were analyzed using GraphPad Prism version 9.3.1, for MAC (GraphPad Software, La Jolla California US; www.graphpad.com). Figures were created using *R* v. 4.1.0 (https://www.r-project.org). The Student *t* tests and Fisher exact tests were used to assess statistical differences in clinical features between the IgA nephropathy and RT groups. Regarding relative abundance comparisons, TCR repertoire-sequencing analysis of the TRAV, J, and TRBV; J gene segments; CDR3 amino acids; diversity index; and similarity index were evaluated using the Mann–Whitney *U* test. We also investigated the correlation between the expression levels of the TCR repertoire and tonsillar galactose-deficient IgA1 (Gd-IgA1) levels, clinical characteristics, and the IgA index in the tonsils, as previously reported by our group.^5^ Spearman rank correlation coefficient was used for correlation analysis. To mitigate the risk of type 1 error from multiple comparisons in this exploratory analysis, *P* values were adjusted using the Benjamini-Hochberg false discovery rate method where appropriate, and an adjusted *P* value < 0.05 was used to determine statistical significance for these multiple comparisons. Furthermore, to indicate the magnitude and precision of the observed effects, effect sizes (*e.g*., Hodges-Lehmann estimated median difference [ΔHL] for Mann–Whitney *U* tests) and their corresponding 95% confidence intervals (CIs) were calculated and reported alongside *P* values.

## Results

### Clinical Characteristics of Enrolled Patients

The patients' clinical characteristics are listed in Table 1. Samples from our previous study were used in this study.^5^ Significant differences were found between the two groups in the levels of total protein and ratio of renin-angiotensin inhibitor use.

**Table 1:** Characteristics of enrolled patients

Patient Characteristics	IgA nephropathy *n*=27	RT *n*=20	*P* Value
Age (yr)	31.9±10.7	30.3±10.4	0.74
Sex (male)	12 (44.4)	12 (60.0)	0.38
BMI (kg/m^2^)	21.1±2.1	22.6±5.0	0.38
Systolic BP (mm Hg)	111.9±10.2	116.5±13.6	0.33
Diastolic BP (mm Hg)	68.4±7.6	70.7±11.4	0.62
S-Cre (mg/dl)	0.84±0.26	0.72±0.15 (*n*=19)	0.18
eGFR (ml/min per 1.73 m^2^)	82.0±24.7	97.35±20.9	0.063
BUN (mg/ml)	12.5±4.8	12.3±3.2	0.64
TP (g/dl)	7.2±0.5 (*n*=26)	7.5±0.5 (*n*=14)	0.033
UA (mg/dl)	5.4±1.3	5.2±1.4 (*n*=8)	0.77
IgA (mg/dl)	292.1±119.0	—	—
C3 (mg/dl)	90.1±21.5 (*n*=24)	—	—
Urinary protein (g/d)	0.52±0.32 (*n*=27)	—	—
RASI	17 (62.9)	0 (0)	<0.001
**Oxford classification (*n*=26)**			
Mesangial hypercellularity (M0/M1)	22/4		
Endocapillary hypercellularity (E0/E1)	12/14		
Segmental glomerulosclerosis (S0/S1)	4/22		
Tubular atrophy/interstitial fibrosis (T0/T1/T2)	26/0/0		

Data are presented as the means±SD, median (interquartile range), or number (ratio) and were statistically compared using the Student *t* test or Fisher exact test. Among the clinical characteristics of these patients, significant differences were noted in serum creatinine, eGFR, and renin-angiotensin-aldosterone system inhibitor scores.

BMI, body mass index; C3, complement component 3; RASI, renin-angiotensin-aldosterone system inhibitor; RT, recurrent tonsillitis; s-Cre, serum creatinine; TP, total protein; UA, uric acid.

### Diversity and Similarity of TCR Repertoire between Patients with IgA nephropathy and Those with RT

**Figure 2:**
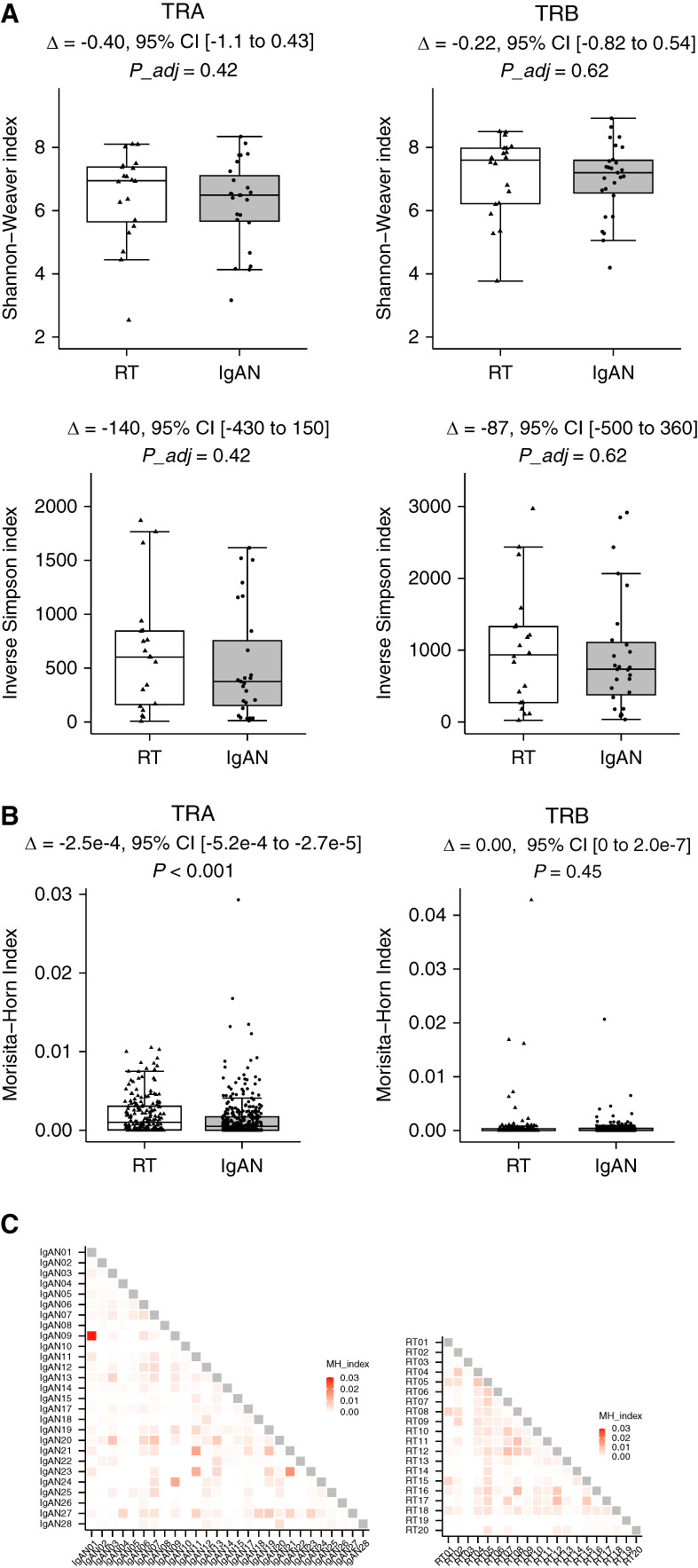
**Diversity and similarity of TRA and TRB repertoires in patients with IgA nephropathy and patients with RT.** (A) Shannon-Weaver index and inverse Simpson index were used as indices of diversity. Diversity indexes of Shannon and inversion Simpson in TCR clones in patients with IgA nephropathy (*n*=27) and patients with RT (*n*=20). Group differences were assessed using the Mann–Whitney *U* test with FDR correction (Benjamini-Hochberg method) for multiple comparisons. Data are shown as median and IQR (Mann–Whitney *U* test). Reported statistics include effect sizes (Δ), 95% CI, and *P* values adjusted for multiple comparisons. (B) Morisita-Horn index, index of TRA and TRB similarity, was calculated with R program and compared. Data are shown as median and IQR. Reported statistics include effect sizes (Δ), 95% CI, and *P* values. (C) Heatmap of Morisita-Horn index in the TRA repertoire in each group. CI, confidence interval; FDR, false discovery rate; IQR, interquartile range.

To evaluate TCR diversity, the Shannon-Weaver and Inverse Simpson indices were calculated and normalized to minimize the influence of sequencing depth (Figure 2A). Both indices failed to show any significant differences between RT and IgA nephropathy patients. V/J segment usage was also compared between both groups, and no significant differences between the groups were observed (Supplemental Figures 1 and 2). However, the Morisita-Horn index, calculated as a similarity index between the groups, was significantly higher (*P* < 0.001) for TRA repertoires in patients with RT than in those with IgA nephropathy (ΔHL=−2.5e-4, 95% CI [−5.2e-4 to −2.7e-5], *P* < 0.001; Figure 2B). When the similarities among the patients with IgA nephropathy and those with RT were graphically presented, the index densities of patients with IgA nephropathy were sparser than those of patients with RT (Figure 2C). These results indicated that the TRA repertoires in patients with RT are shared more as a whole than in patients with IgA nephropathy.

### TCRs Shared among Patients with IgA nephropathy Frequently Have Shorter CDR3 Length

**Figure 3:**
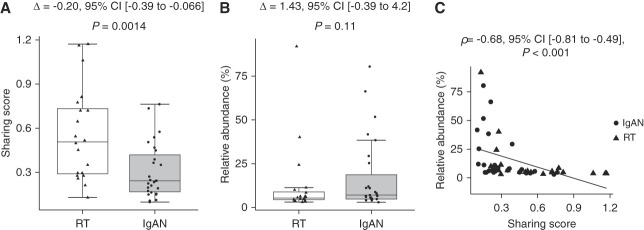
**Sharing states and relative abundance of public TRA repertoires shared by more than two samples from patients with IgA nephropathy and RT cases.** (A) Sharing states of TRA clonotypes shared among patients in both groups. Sharing scores were determined by the ratio of the number of shared TRA clonotypes in each sample against the number of all shared TRA clonotypes. The difference in the distribution of sharing scores between patients with IgA nephropathy (*n*=27) and those with RT (*n*=20) was statistically assessed using the Mann–Whitney *U* test. Data are shown as median and IQR. Reported statistics include effect sizes (Δ), 95% CI, and *P* values. (B) Comparison of the relative abundance of public TRA repertoires between patients with IgA nephropathy (*n*=27) and those with RT (*n*=20). Data are shown as median and IQR using the Mann–Whitney *U* test. Reported statistics include effect sizes (Δ), 95% CI, and *P* values. (C) Correlation between sharing scores and relative abundance of shared TRA repertoire. The data on sharing scores and relative abundance of shared TRA repertoire in patients with IgA nephropathy (*n*=27) and those with RT (*n*=20) were statistically evaluated through Spearman correlation and linear regression analysis. The solid line represents the linear regression line. Reported statistics include effect sizes, 95% CI, and *P* values.

To focus on shared TRA repertoires, we used TRA clones with more than two reads and shared with more than two patients in each group. To evaluate the number of shared TRA repertoires, we calculated sharing scores and found them to be significantly lower (ΔHL=−0.20, 95% CI [−0.39 to −0.066], *P* = 0.0014) in the IgA nephropathy than in the RT groups (Figure 3A). However, the relative abundance of the shared repertoire was higher (ΔHL=1.4, 95% CI [−0.39 to 4.2], *P* = 0.11) in the IgA nephropathy group (Fig 3B). Among these, the relative abundance of repertoires shared between two (ΔHL=1.2, 95% CI [−0.10 to 3.2], *P_adj* = 0.13) and three samples (ΔHL=0.26, 95% CI [−0.073 to 0.55], *P_adj* = 0.13) tended to be higher in the IgA nephropathy than in the RT group (Table 2 and Supplemental Figure 3). In addition, sharing scores were significantly negatively correlated with the relative abundance of the shared TRA repertoire (ρ=−0.68, 95% CI [−0.81 to −0.49], *P* < 0.001; Figure 3C).

**Table 2:** Public *T*-cell receptor clonotypes shared among each group

Shared Samples	IgA nephropathy (*n*=27)		RT (*n*=20)	
	Observed Clonotypes	Relative Abundance	Observed Clonotypes	Relative Abundance
9	0	1.5	2	1.3
8	3		0	
7	1		0	
6	3		2	
5	13		7	
4	21	1.8	14	1.1
3	78	2.6	56	1.7
2	672	10.2	461	6.1
1	33,753	83.7	26,120	89.5

Shared samples: the number indicating how many samples were shared. Observed clonotype: number of clonotypes observed in each group. Relative abundance: relative abundance of clonotypes observed in each shared sample (%).

RT, recurrent tonsillitis.

**Figure 4:**
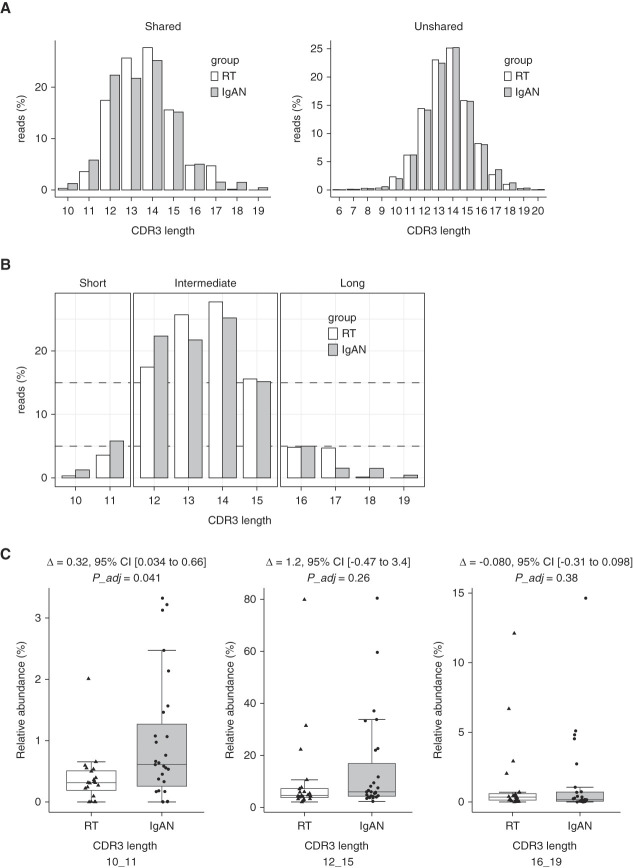
**Distribution of CDR3 length in public TRA repertoires and relative abundance of public TRA repertoires with short CDR3 length.** (A) CDR3 length calculated with shared TRA (left) and private TRA (right, unshared). The percentage frequency of reads in each CDR3 length in shared TRA repertoires (left, *n*=27) and in private TRA repertoires (right, *n*=20) is shown as a bar graph. (B) Based on this distributional pattern, CDR3 lengths were stratified into three bins (10–11, 12–15, and 16–19 amino acids) for subsequent analyses. (C) Comparison between the relative abundance of TRA repertoires in each bins; short (10–11 amino acids, left), intermediate (12–15 amino acids, central), and long (16–19 amino acids, right) between patients with RT (*n*=20) and those with IgA nephropathy (*n*=27). Box plots show median, IQR with whiskers of 1.5 x IQR, and statistically compared using the Mann–Whitney *U* test with FDR correction (Benjamini-Hochberg method) for multiple comparisons. Reported statistics include effect sizes (Δ), 95% CI, and *P* values adjusted for multiple comparisons.

As shown in Figure 4A, the unshared TRA repertoires showed different CDR3 length distributions between the two groups. CDR3 length distribution of shared clonotypes showed a non-uniform, trimodal pattern, with a central enrichment around 12–15 amino acids and relative depletion at shorter (ten to 11) and longer (16–19) lengths. Based on this distribution pattern, CDR3 lengths were stratified into three bins (ten to 11, 12–15, and 16–19 amino acids) for subsequent analyses (Figure 4B). Compared with patients with RT, patients with IgA nephropathy exhibited significantly increased relative abundances of TRA clonotypes with CDR3 lengths of short bins (ΔHL=0.32, 95% CI [0.034 to 0.66], *P_adj* = 0.041, Figure 4C, left); however, no significant differences between groups were observed in intermediate and long bins. Then, we defined shared TRA clonotypes in short bins as IgA nephropathy-associated TRAs.

### Features and Hydrophobicity of TRA Clonotypes Abundant in Patients with IgA Nephropathy

**Figure 5:**
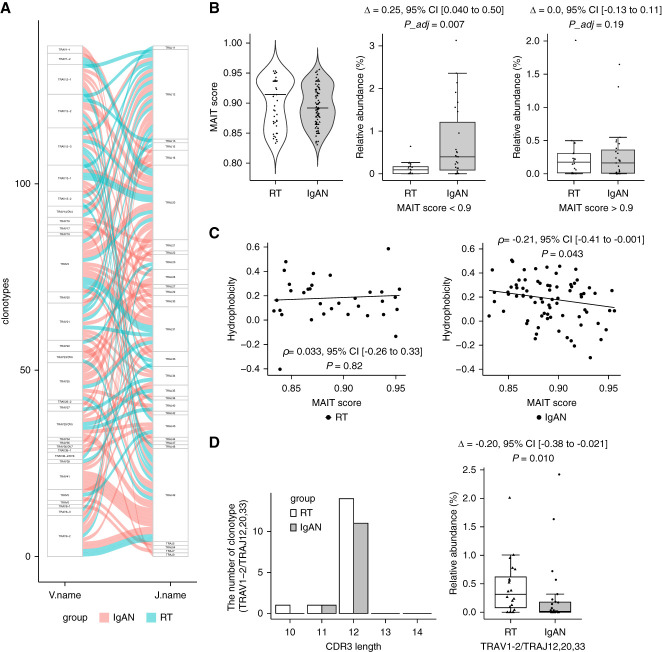
**TRAV/TRAJ gene combination and features of CDR3 amino acids in IgA nephropathy-associated TRAs.** (A) Alluvial diagram of IgA nephropathy-associated TRAs. The combination of TRAV and TRAJ genes in patients with IgA nephropathy (pink) and those with RT (light green) is shown. (B) Distribution of MAIT match scores of IgA nephropathy-associated TRAs repertoires. The relative abundance of IgA nephropathy-associated TRAs of MAIT scores<0.9 (central) and those >0.9 (right) were statistically compared using the Mann–Whitney *U* test with FDR correction (Benjamini-Hochberg method) for multiple comparisons. Reported statistics include effect sizes (Δ), 95% CI, and *P* values adjusted for multiple comparisons. (C) Correlation between MAIT match scores and hydrophobicities of IgA nephropathy-associated TRAs repertoires in patients with RT (left) and those with IgA nephropathy (right). Correlation data are statistically compared using Spearman correlation. Reported statistics include effect sizes, 95% CI, and *P* values. (D) CDR3 length distribution of the number of TRA clonotypes defined by TRAV1-2/TRAJ12,20,33 (left) and the comparison of the relative abundance of TRAV1-2/TRAJ12,20,33 in patients with RT (*n*=20) and those with IgA nephropathy (*n*=27) (right). Box plots show median, IQR with whiskers of 1.5× IQR, and are statistically compared using the Mann–Whitney *U* test. Reported statistics include effect sizes (Δ), 95% CI, and *P* values. TRAJ, *T* cell receptor alpha joining; TRAV, *T* cell receptor alpha variable.

TRAV and TRAJ gene usage in the TRA clonotypes is shown in Figure 5A. The IgA nephropathy-associated TRAs included 13 clones with identical TRAV/TRAJ gene usage and CDR3 amino acid sequences shared between both groups: TRAV12-3/TRAJ12, TRAV13-2/TRAJ12, TRAV14/DV4/TRAJ12, TRAV2/TRAJ12, TRAV20/TRAJ12, TRAV21/TRAJ12, TRAV38-2/DV8/TRAJ12, TRAV9-2/TRAJ12, TRAV1-2/TRAJ20, TRAV2/TRAJ31, TRAV25/TRAJ31, TRAV29/DV5/TRAJ35, and TRAV12-2/TRAJ49. All other clonotypes were unique to each group.

Shared TRA repertoires, such as MAIT cells, were observed in invariant *T* cells. Patients with IgA nephropathy had a higher number of TRA clonotypes with low MAIT scores (<0.9) than did those with RT (Figure 5B, left). The relative abundance of those with low MAIT was significantly higher in patients with IgA nephropathy than in those with RT (ΔHL=0.25, 95% CI [0.04 to 0.50], *P_adj* = 0.007, Figure 5B, center), whereas those with high MAIT did not differ (ΔHL=0.0, 95% CI [−0.13 to 0.11], *P_adj* = 0.19, Figure 5B, right). The hydrophobicities of IgA nephropathy-associated TRAs did not differ between the groups (Supplemental Figure 4); however, they negatively correlated with MAIT scores in patients with IgA nephropathy (ρ=−0.21, 95% CI [−0.41 to −0.001], *P_adj* = 0.043, Figure 5C, right), but not in RT cases (ρ=0.033, 95% CI [−0.26 to 0.33], *P_adj* = 0.82). When this correlation was analyzed using the clonotypes found only in patients with IgAN, we found similar statistical significance in the correlation between MAIT scores and hydrophobicities in patients with IgA nephropathy (Supplemental Figure 5).

Most TRAs of MAIT cells (TRAV1-2/TRAJ12,20,33) had a CDR3 length of 12 amino acids (Figure 5D, left). The relative abundances of TRAV1-2/TRAJ12,20,33 showed significantly increased levels in the RT compared with the IgA nephropathy groups (ΔHL=−0.20, 95% CI [−0.38 to −0.021], *P* = 0.010; Figure 5D, right).

To further characterize IgA nephropathy-associated TRAs, we searched public TCR databases (VDJdb, UcTCRdb, and pathology-associated TCR database) for matching sequences. Twenty of 90 IgA nephropathy-associated TRA clonotypes (22.2%) were found in at least one of these databases. Notably, 13 clonotypes (14.4%) were annotated as CD8*αα*-associated in UcTCRdb (Supplemental Table 4). No clonotypes matched sequences annotated as MAIT-cell TCRs in UcTCRdb. Overall, 70 clonotypes (77.8%) did not match any entries in these databases.

### Correlation of IgA nephropathy-Associated TRAs with *Bacteroidetes* IgA-Binding Index and Gd-IgA1 Levels

**Figure 6:**
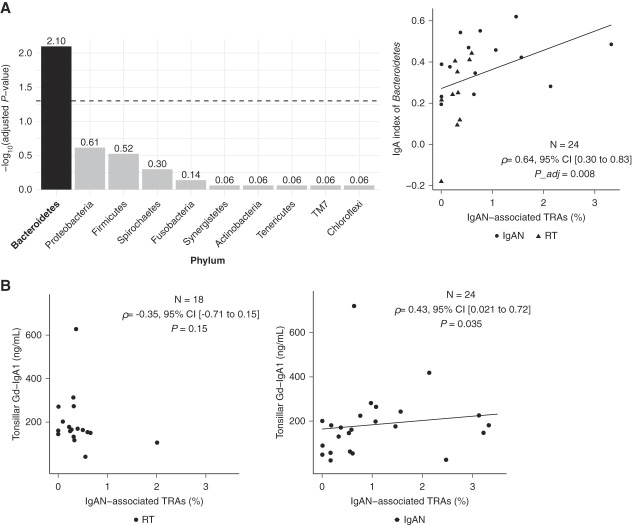
**Correlations of IgA nephropathy-associated TRAs with bacterial IgA-binding index and tonsillar Gd-IgA1.** (A) Correlations between the relative abundance of IgA nephropathy-associated TRAs and IgA binding indices of bacteria at tonsils in patients of both groups (*n*=24) were statistically assessed using Spearman correlation and linear regression analysis. The *P* values were adjusted using the Benjamini and Hochberg method. The left panel shows −log10 (adjusted *P* values), with phylum Bacteroidetes highlighted in black. A dotted line indicates the level of significance for the adjusted *P* value. Correlation between the relative abundance of IgA nephropathy-associated TRAs and IgA binding index of Bacteroidetes at tonsils (right). (B) Correlation between the relative abundance of IgA nephropathy-associated TRAs and tonsillar IgA1 levels in RT (left, *n*=18) and IgA nephropathy patients (right, *n*=24). Correlation data were statistically compared using Spearman correlation and linear regression analysis. The solid line represents the linear regression line. Reported statistics include effect sizes, 95% CI, and *P* values.

The IgA-binding index of the phylum Bacteroidetes significantly positively correlated with the relative abundance of IgA nephropathy-associated TRAs (ρ=0.64, 95% CI [0.30 to 0.83], adjusted *P_adj* = 0.008, Figure 6A). None of the other bacterial phyla and genera showed a significant correlation with these measures (Figure 6A and Supplemental Figure 7). We also identified a significant and positive association between tonsillar Gd-IgA1 concentrations and the frequency of IgA nephropathy-associated TRAs in the IgA nephropathy group, which was not observed in patients with RT (ρ=0.43, 95% CI [0.021 to 0.72], *P* = 0.035; Figure 6B).

## Discussion

This study demonstrated that the TRA repertoire exhibited significantly lower similarity indices in patients with IgA nephropathy compared with patients with RT, whereas no such difference was observed in the TRB repertoire. Furthermore, shared TRA clonotypes characterized by short CDR3 lengths and low MAIT match scores, defined as IgA nephropathy-associated TRAs, were significantly more abundant in patients with IgA nephropathy than in those with RT, suggesting a distinct expansion of *T* cells harboring invariant TRA chains in patients with IgA nephropathy. The expression of these TRA clonotypes was significantly correlated with the IgA binding index of the phylum Bacteroidetes, to which tonsillar and serum IgA in patients with IgA nephropathy preferentially bind.^5^ Moreover, those clonotypes correlated with tonsillar Gd-IgA1 levels in patients with IgA nephropathy, which were associated with Gd-IgA1 deposition in glomeruli and mesangial cell proliferation.^5^ The aberrant *T*-cell subsets revealed in this study may be involved in the abnormal immune reactions of the tonsils during IgA nephropathy development.

We also found differences in the similarity of TRA repertoires between patients with IgA nephropathy and those with RT demonstrating significant expression of shared TRA clonotypes with short CDR3 lengths in patients with IgA nephropathy. Public TCRs are observed in the fields of infection, malignant tumors, and autoimmunity.^26^ *T* cells bearing public TCRs represent MAIT cells and invariant natural killer *T* cells, among others.^27^ Distinct *T* cells harboring public TRAs play a role in disease development. A semi-invariant TRA has been observed in the peripheral blood *T* cells of patients with Crohn's disease.^13^ In patients with type 1 diabetes, public TRA expressed in human islet-reactive *T* cells has been observed in the peripheral blood.^14,15^ Recently, Gentile *et al*. reported that patients with IgA nephropathy exhibit a lower number of MAIT cells in their peripheral blood than do healthy individuals.^28^ However, blood levels of double-negative *T* cells were increased, suggesting that unconventional *T* cells play a role in the pathophysiology of IgA nephropathy. Similarly, our study showed a significant usage of shared TRA characterized by short CDR3 length and low MAIT match scores in the tonsils of patients with IgA nephropathy. By contrast, the abundance of MAIT-type TRAs was significantly lower in patients with IgA nephropathy than in those with RT. In addition, the public TCR databases did not annotate any IgA nephropathy-associated TRAs as MAIT cell-associated, although these annotations are limited by database coverage. This leads us to hypothesize that a subset of unconventional *T* cells other than MAIT cells may play a distinct role in mucosal immunity in patients with IgA nephropathy; however, this hypothesis requires validation using orthogonal experimental approaches.

The IgA nephropathy-associated TRAs repertoire was characterized by a short CDR3 length and limited gene usage, potentially involved in TCR recombination bias, which is the preferential usage of limited germline genes in recombination events for TCR. Germline-encoded genes, such as TRAV41,^29^ are significantly enriched at the CDR3 junction of IgA nephropathy-associated TRAs, coupled with the preferential use of TRAJ49 in patients with IgA nephropathy. TCRs with these recombination biases are involved in innate immunity.^30^ Public TRA with short CDR3 lengths and rich hydrophobic clonotypes are associated with autoimmunity.^14^ We anticipated that TRA clonotypes from germline-encoded genes in the tonsils of patients with IgA nephropathy would react aberrantly with antigens as part of their innate immune response. Indeed, we found a significant correlation between IgA nephropathy-associated TRAs and the IgA-binding index of bacteroidetes, which has been previously reported to be higher in patients with IgA nephropathy than in those with RT.^5^ In addition, a significant negative correlation between MAIT match scores and hydrophobicities of IgA nephropathy-associated TRAs was observed in patients with IgA nephropathy, suggesting that they have a restricted configuration and degenerative recognition capacity against a distinct antigen environment.

IgA nephropathy-associated TRAs may play a pivotal role in the aberrant mucosal immunity in IgA nephropathy cases.

Significant correlations between IgA nephropathy-associated TRAs and tonsillar Gd-IgA1 levels imply a relationship between *T*-cell immunity and the production of pathogenic IgA. Previously, we demonstrated a significant association between tonsillar Gd-IgA1 levels, glomerular Gd-IgA1 staining, and mesangial cell proliferation, as assessed using the Oxford classification.^5^ The diversity indices of the immunoglobulin heavy constant alpha repertoire significantly correlated with tonsillar Gd-IgA1 levels in patients with IgA nephropathy (Supplemental Figure 6). This suggests that diversely expanded immune cells, coupled with distinct *T* cells harboring IgA nephropathy-associated TRAs, produce pathogenic Gd-IgA1 in the tonsils of patients with IgA nephropathy. Based on the relationship between IgA nephropathy-associated TRAs and IgA binding to bacteria, we anticipate the associated between pathogenic Gd-IgA1 and the microbiome through these distinct TRAs. In CD studies, semi-invariant TRA associated with this disorder have cross-reactive features with the gut microbiome.^31^ Recently, pathogenic IgA antibodies reactive to spectrin and CBX3, which are rich in galactose, have been reported in patients with IgA nephropathy, indicating that an autoimmune process is involved in IgA nephropathy development.^32^ Oral commensal bacteria possess epitopes recognized by the IgA anti-CBX3 antibody.^33^ Unconventional *T* cells, such as MAIT cells, aid in the B-cell response, thereby yielding IgA production.^34^ Therefore, we hypothesized that distinct TRA clonotypes interacting with unique microbiota in tonsils might be involved in IgA nephropathy development through mechanisms of cross-reactivity or molecular mimicry through the production of distinct IgA, such as Gd-IgA1. However, further research is required to confirm this hypothesis.

This study has some limitations. First, we used palatine tonsils from patients with RT as controls because of ethical and practical challenges in obtaining tonsillar tissue from healthy individuals. However, RT is characterized by chronic inflammation and thus does not represent healthy mucosal immunity. Accordingly, the differences observed between the IgA nephropathy and RT groups may reflect differences in inflammatory status or broader disease context rather than IgA nephropathy-specific immune features. Second, this study used bulk TCR-seq and did not characterize *T*-cell subsets using cell-based assays. Our classifications (*e.g*., low MAIT match score clonotypes) should be interpreted as computationally inferred signatures based on TCR sequence features rather than definitive subset identities or functions. In future studies, single-cell TCR-seq coupled with transcriptomic profiling (and/or flow cytometry) revealing the gene segment usage of CDR3 and its cellular gene expression may enable direct mapping of these clonotypes to specific *T*-cell subsets. Third, we were unable to demonstrate a significant correlation between IgA nephropathy-associated TRAs and serum Gd-IgA1 levels because paired serum samples were available for only a limited number of patients (data not shown). Because circulating Gd-IgA1 likely reflects heterogeneous contributions from multiple mucosal sites and systemic compartments, any association with a mucosa-associated *T*-cell signature may be attenuated compared with tonsillar Gd-IgA1. Therefore, a larger cohort will be required to accurately evaluate the relationship between IgA nephropathy-associated TRAs and serum Gd-IgA1.

Although a few studies have focused on the diversity of TCRs in the tonsils^35^ and kidneys^36^ of patients with IgA nephropathy, comprehensive TCR analysis using high-throughput sequencing technology, as in this study, has not been conducted on human tonsillar tissues to date. This thorough sequence analysis helped elucidate the similarities and differences in TRA repertoires and the overexpression of distinct TRAs in patients with IgA nephropathy.

In conclusion, the findings of this study suggest the involvement of aberrant *T* cells in the abnormal immune activity in the tonsillar mucosa of patients with IgA nephropathy.

## Supplementary Material

## Data Availability

Raw individual-level human sequencing data are not publicly available due to informed consent and IRB-approved protocol restrictions. Processed case-level data are available in the manuscript and Supplementary Material. Additional information may be provided upon reasonable request.

## Declarative Statements

This study includes clinical experimentation and received Institutional Review Board or Ethics Committee approval. All patients provided written informed consent. This study includes clinical experimentation and complies with the Declaration of Helsinki. The clinical and research activities being reported are consistent with the Principles of the Declaration of Istanbul as outlined in the Declaration of Istanbul on Organ Trafficking and Transplant Tourism.

## Supplemental Material

Supplemental Table 1. Primers for adaptor-ligation PCR and *T*-cell receptor repertoire.

Supplemental Table 2. Numbers of total reads, in-frame reads, and in-frame unique reads obtained in TRA and TRB repertoire sequencing of tonsillar samples.

Supplemental Table 3. Summary of public and private TRA and TRB repertoire in IgA nephropathy and RT tonsils.

Supplemental Table 4. Summary of clonotypes in IgA nephropathy-associated TRAs matching public TCR databases.

Supplemental Figure 1. Comparison of the relative abundance of TRAV and TRAJ with in-frame clones in patients with IgA nephropathy and patients with RT.

Supplemental Figure 2. Comparison of the relative abundance of TRBV and *T* cell receptor beta joining with in-frame clones in patients with IgA nephropathy and patients with RT.

Supplemental Figure 3. Comparison of the relative abundances of public TRA repertoires shared between two, three, and more than four samples from patients with IgA nephropathy and patients with RT.

Supplemental Figure 4. Comparison of the hydrophobicity of IgA nephropathy-associated TRAs in patients with IgA nephropathy and patients with RT.

Supplemental Figure 5. Features of CDR3 amino-acid composition in clonotypes uniquely observed in patients with IgA nephropathy and comparison of patients with IgA nephropathy and patients with RT.

Supplemental Figure 6. Correlations between tonsillar Gd-IgA1 levels and the diversity indices of the immunoglobulin heavy constant alpha repertoire in tonsils in patients of both groups.

Supplemental Figure 7. Spearman correlation analysis performed to assess the association between the relative abundance of IgA nephropathy-associated TRAs and the IgA-binding index of *Bacteroidetes* genera in the tonsils.
