# Supplementary material for: Shared T-Cell Receptor Repertoire in the Tonsils of Patients with IgA Nephropathy
Source: Kidney360. 2026 Feb 10;7(7):1503–15. doi: 10.34067/KID.0000001154 (PMC13450961; doi:10.34067/KID.0000001154)
Supplement: Supplementary file 1 [file kidney360-7-1503-s001.pdf]

ASN American Society of Nephrology
JASN Journal of the American Society of Nephrology
KIDNEY360 Accessing Our World From Every Angle
CJASN Clinical Journal of the American Society of Nephrology

ASN Journal Disclosure Form

As per ASN journal policy, I have disclosed any financial relationships or commitments I have held in the past 36 months as included below. I have listed my Current Employer below to indicate there is a relationship requiring disclosure. If no relationship exists, my Current Employer is not listed.

S. Goto reports the following:
Advisory or Leadership Role: Novartis Pharma, Otsuka Pharmaceutical

I understand that the information above will be published within the journal article, if accepted, and that failure to comply and/or to accurately and completely report the potential financial conflicts of interest could lead to the following: 1) Prior to publication, article rejection, or 2) Post-publication, sanctions ranging from, but not limited to, issuing a correction, reporting the inaccurate information to the authors' institution, banning authors from submitting work to ASN journals for varying lengths of time, and/or retraction of the published work.

Name: Shin Goto
Manuscript ID: K360-2025-001226R1
Manuscript Title: Shared T-Cell Receptor Repertoire in the Tonsils of Patients with Immunoglobulin A Nephropathy
Date of Completion: January 9, 2026
Disclosure Updated Date: January 9, 2026

ASN American Society of Nephrology | JASN Journal of the American Society of Nephrology | KIDNEY360 Accessing Our World From Every Angle | CJASN Clinical Journal of the American Society of Nephrology

ASN Journal Disclosure Form

As per ASN journal policy, I have disclosed any financial relationships or commitments I have held in the past 36 months as included below. I have listed my Current Employer below to indicate there is a relationship requiring disclosure. If no relationship exists, my Current Employer is not listed.

K. Higashi reports the following:
Employer: National Institute of Genetics

I understand that the information above will be published within the journal article, if accepted, and that failure to comply and/or to accurately and completely report the potential financial conflicts of interest could lead to the following: 1) Prior to publication, article rejection, or 2) Post-publication, sanctions ranging from, but not limited to, issuing a correction, reporting the inaccurate information to the authors' institution, banning authors from submitting work to ASN journals for varying lengths of time, and/or retraction of the published work.

Name: Koichi Higashi
Manuscript ID: K360-2025-001226R1
Manuscript Title: Shared T-Cell Receptor Repertoire in the Tonsils of Patients with Immunoglobulin A Nephropathy
Date of Completion: January 13, 2026
Disclosure Updated Date: January 13, 2026

ASN American Society of Nephrology JASN Journal of the American Society of Nephrology KIDNEY360 Accessing Our World From Every Angle CJASN Clinical Journal of the American Society of Nephrology

ASN Journal Disclosure Form

As per ASN journal policy, I have disclosed any financial relationships or commitments I have held in the past 36 months as included below. I have listed my Current Employer below to indicate there is a relationship requiring disclosure. If no relationship exists, my Current Employer is not listed.

A. Horii reports the following:
Employer: Niigata University

I understand that the information above will be published within the journal article, if accepted, and that failure to comply and/or to accurately and completely report the potential financial conflicts of interest could lead to the following: 1) Prior to publication, article rejection, or 2) Post-publication, sanctions ranging from, but not limited to, issuing a correction, reporting the inaccurate information to the authors' institution, banning authors from submitting work to ASN journals for varying lengths of time, and/or retraction of the published work.

Name: Arata Horii
Manuscript ID: K360-2025-001226R1
Manuscript Title: Shared T-Cell Receptor Repertoire in the Tonsils of Patients with Immunoglobulin A Nephropathy
Date of Completion: January 10, 2026
Disclosure Updated Date: January 10, 2026

ASN Journal Disclosure Form

As per ASN journal policy, I have disclosed any financial relationships or commitments I have held in the past 36 months as included below. I have listed my Current Employer below to indicate there is a relationship requiring disclosure. If no relationship exists, my Current Employer is not listed.

Y. Kaneko reports the following:
Employer: Niigata University Graduate School of Medical and Dental Sciences; and Patents or Royalties: Momenta Pharmaceuticals, Inc.

I understand that the information above will be published within the journal article, if accepted, and that failure to comply and/or to accurately and completely report the potential financial conflicts of interest could lead to the following: 1) Prior to publication, article rejection, or 2) Post-publication, sanctions ranging from, but not limited to, issuing a correction, reporting the inaccurate information to the authors' institution, banning authors from submitting work to ASN journals for varying lengths of time, and/or retraction of the published work.

Name: Yoshikatsu Kaneko
Manuscript ID: K360-2025-001226R1
Manuscript Title: Shared T-Cell Receptor Repertoire in the Tonsils of Patients with Immunoglobulin A Nephropathy
Date of Completion: January 13, 2026
Disclosure Updated Date: January 9, 2026

ASN
American Society of Nephrology
JASN
Journal of the American Society of Nephrology
KIDNEY360
Accessing Our World From Every Angle
CJASN
Clinical Journal of the American Society of Nephrology

ASN Journal Disclosure Form

As per ASN journal policy, I have disclosed any financial relationships or commitments I have held in the past 36 months as included below. I have listed my Current Employer below to indicate there is a relationship requiring disclosure. If no relationship exists, my Current Employer is not listed.

K. Kurokawa reports the following:
Research Funding: TOYOTA MOTOR CORPORATION

I understand that the information above will be published within the journal article, if accepted, and that failure to comply and/or to accurately and completely report the potential financial conflicts of interest could lead to the following: 1) Prior to publication, article rejection, or 2) Post-publication, sanctions ranging from, but not limited to, issuing a correction, reporting the inaccurate information to the authors' institution, banning authors from submitting work to ASN journals for varying lengths of time, and/or retraction of the published work.

Name: Ken Kurokawa
Manuscript ID: K360-2025-001226R1
Manuscript Title: Shared T-Cell Receptor Repertoire in the Tonsils of Patients with Immunoglobulin A Nephropathy
Date of Completion: January 10, 2026
Disclosure Updated Date: January 10, 2026

ASN American Society of Nephrology | JASN Journal of the American Society of Nephrology | KIDNEY360 Accessing Our World From Every Angle | CJASN Clinical Journal of the American Society of Nephrology

ASN Journal Disclosure Form

As per ASN journal policy, I have disclosed any financial relationships or commitments I have held in the past 36 months as included below. I have listed my Current Employer below to indicate there is a relationship requiring disclosure. If no relationship exists, my Current Employer is not listed.

I. Narita reports the following:
Research Funding: Otsuka Pharmaceutical, Kyowa-kirin, Daiichi-Sankyo, Sumitomo Pharma, Terumo, Bayer, Astellas Pharma, Sumitomo Pharma, Ono Pharmaceutical, Amicus Thepeuticus; and Honoraria: Kyowa-Kirin, Otsuka Pharmaceutical, Sanofi, Bayer.

I understand that the information above will be published within the journal article, if accepted, and that failure to comply and/or to accurately and completely report the potential financial conflicts of interest could lead to the following: 1) Prior to publication, article rejection, or 2) Post-publication, sanctions ranging from, but not limited to, issuing a correction, reporting the inaccurate information to the authors' institution, banning authors from submitting work to ASN journals for varying lengths of time, and/or retraction of the published work.

Name: Ichiei Narita
Manuscript ID: K360-2025-001226R1
Manuscript Title: Shared T-Cell Receptor Repertoire in the Tonsils of Patients with Immunoglobulin A Nephropathy
Date of Completion: January 9, 2026
Disclosure Updated Date: January 9, 2026

ASN
American Society of Nephrology
JASN
Journal of the American Society of Nephrology
KIDNEY360
Accessing Our World From Every Angle
CJASN
Clinical Journal of the American Society of Nephrology

ASN Journal Disclosure Form

As per ASN journal policy, I have disclosed any financial relationships or commitments I have held in the past 36 months as included below. I have listed my Current Employer below to indicate there is a relationship requiring disclosure. If no relationship exists, my Current Employer is not listed.

K. Satokata reports the following:
Employer: Niigata University

I understand that the information above will be published within the journal article, if accepted, and that failure to comply and/or to accurately and completely report the potential financial conflicts of interest could lead to the following: 1) Prior to publication, article rejection, or 2) Post-publication, sanctions ranging from, but not limited to, issuing a correction, reporting the inaccurate information to the authors' institution, banning authors from submitting work to ASN journals for varying lengths of time, and/or retraction of the published work.

Name: Kazunori Satokata
Manuscript ID: K360-2025-001226R1
Manuscript Title: Shared T-Cell Receptor Repertoire in the Tonsils of Patients with Immunoglobulin A Nephropathy
Date of Completion: January 10, 2026
Disclosure Updated Date: January 10, 2026

ASN Journal Disclosure Form

As per ASN journal policy, I have disclosed any financial relationships or commitments I have held in the past 36 months as included below. I have listed my Current Employer below to indicate there is a relationship requiring disclosure. If no relationship exists, my Current Employer is not listed.

N. Takahashi has nothing to disclose.

I understand that the information above will be published within the journal article, if accepted, and that failure to comply and/or to accurately and completely report the potential financial conflicts of interest could lead to the following: 1) Prior to publication, article rejection, or 2) Post-publication, sanctions ranging from, but not limited to, issuing a correction, reporting the inaccurate information to the authors' institution, banning authors from submitting work to ASN journals for varying lengths of time, and/or retraction of the published work.

Name: Nao Takahashi
Manuscript ID: K360-2025-001226R1
Manuscript Title: Shared T-Cell Receptor Repertoire in the Tonsils of Patients with Immunoglobulin A Nephropathy,
Date of Completion: January 13, 2026
Disclosure Updated Date: January 13, 2026

ASN American Society of Nephrology
JASN Journal of the American Society of Nephrology
KIDNEY360 Accessing Our World From Every Angle
CJASN Clinical Journal of the American Society of Nephrology

ASN Journal Disclosure Form

As per ASN journal policy, I have disclosed any financial relationships or commitments I have held in the past 36 months as included below. I have listed my Current Employer below to indicate there is a relationship requiring disclosure. If no relationship exists, my Current Employer is not listed.

H. Watanabe has nothing to disclose.

I understand that the information above will be published within the journal article, if accepted, and that failure to comply and/or to accurately and completely report the potential financial conflicts of interest could lead to the following: 1) Prior to publication, article rejection, or 2) Post-publication, sanctions ranging from, but not limited to, issuing a correction, reporting the inaccurate information to the authors' institution, banning authors from submitting work to ASN journals for varying lengths of time, and/or retraction of the published work.

Name: Hirofumi Watanabe
Manuscript ID: K360-2025-001226R1
Manuscript Title: Shared T-Cell Receptor Repertoire in the Tonsils of Patients with Immunoglobulin A Nephropathy
Date of Completion: January 9, 2026
Disclosure Updated Date: January 9, 2026

ASN
American Society of Nephrology
JASN
Journal of the American Society of Nephrology
KIDNEY360
Accessing Our World From Every Angle
CJASN
Clinical Journal of the American Society of Nephrology

ASN Journal Disclosure Form

As per ASN journal policy, I have disclosed any financial relationships or commitments I have held in the past 36 months as included below. I have listed my Current Employer below to indicate there is a relationship requiring disclosure. If no relationship exists, my Current Employer is not listed.

H. Yamaguchi has nothing to disclose.

I understand that the information above will be published within the journal article, if accepted, and that failure to comply and/or to accurately and completely report the potential financial conflicts of interest could lead to the following: 1) Prior to publication, article rejection, or 2) Post-publication, sanctions ranging from, but not limited to, issuing a correction, reporting the inaccurate information to the authors' institution, banning authors from submitting work to ASN journals for varying lengths of time, and/or retraction of the published work.

Name: Hiroki Yamaguchi
Manuscript ID: K360-2025-001226R1
Manuscript Title: Shared T-Cell Receptor Repertoire in the Tonsils of Patients with Immunoglobulin A Nephropathy
Date of Completion: January 10, 2026
Disclosure Updated Date: January 10, 2026

ASN
American Society of Nephrology
JASN
Journal of the American Society of Nephrology
KIDNEY360
Accessing Our World From Every Angle
CJASN
Clinical Journal of the American Society of Nephrology

ASN Journal Disclosure Form

As per ASN journal policy, I have disclosed any financial relationships or commitments I have held in the past 36 months as included below. I have listed my Current Employer below to indicate there is a relationship requiring disclosure. If no relationship exists, my Current Employer is not listed.

S. Yamamoto reports the following:
Employer: Niigata University; Research Funding: Toray Medical Co., Ltd; Kaneka Medix Co., Ltd; and Speakers Bureau: Sanwa Kagaku Kenkyusho, Kyowa Kirin Co, Kaneka Medix, FUSO Pharmaceutical Industries, Kissei Pharmaceutical, Ono Pharmaceutical.

I understand that the information above will be published within the journal article, if accepted, and that failure to comply and/or to accurately and completely report the potential financial conflicts of interest could lead to the following: 1) Prior to publication, article rejection, or 2) Post-publication, sanctions ranging from, but not limited to, issuing a correction, reporting the inaccurate information to the authors' institution, banning authors from submitting work to ASN journals for varying lengths of time, and/or retraction of the published work.

Name: Suguru Yamamoto
Manuscript ID: K360-2025-001226R1
Manuscript Title: Shared T-Cell Receptor Repertoire in the Tonsils of Patients with Immunoglobulin A Nephropathy
Date of Completion: January 21, 2026
Disclosure Updated Date: January 21, 2026
