# Supplementary material for: Shared T-Cell Receptor Repertoire in the Tonsils of Patients with IgA Nephropathy
Source: Kidney360. 2026 Feb 10;7(7):1503–15. doi: 10.34067/KID.0000001154 (PMC13450961; doi:10.34067/KID.0000001154)
Supplement: Supplementary file 2 [file kidney360-7-1503-s002.pdf]

Supplemental data

Supplemental Table 1. Primers for adaptor-ligation PCR and T-cell receptor repertoire

Supplemental Table 2. Numbers of total reads, in-frame reads, and in-frame unique reads obtained in TRA and TRB repertoire sequencing of tonsillar samples

Supplemental Table 3. Summary of public and private TRA and TRB repertoire in IgAN and RT tonsils

Supplemental Table 4. Summary of clonotypes in IgAN-associated TRAs matching public TCR databases

Supplemental Figure 1. Comparison of the relative abundance of TRAV and TRAJ with in-frame clones in patients with IgAN and patients with RT

Supplemental Figure 2. Comparison of the relative abundance of TRBV and TRBJ with in-frame clones in patients with IgAN and patients with RT

Supplemental Figure 3. Comparison of the relative abundances of public TRA repertoires shared between two, three, and more than four samples from patients with IgAN and patients with RT

Supplemental Figure 4. Comparison of the hydrophobicity of IgAN-associated TRAs in patients with IgAN and patients with RT

Supplemental Figure 5. Features of CDR3 amino-acid composition in clonotypes uniquely observed in patients with IgAN and comparison in patients with IgAN and patients with RT

Supplemental Figure 6. Correlations between tonsillar Gd-IgA1 levels and the diversity indices of the IGHA repertoire in tonsils in patients of both groups.

Supplemental Figure 7. Spearman's correlation analysis performed to assess the association between the relative abundance of IgAN-associated TRAs and the IgA-binding index of *Bacteroidetes* genera in the tonsils

Supplemental Table 1: Primers for adaptor-ligation PCR and T-cell receptor repertoire

Primer	Sequence (5′–3′)
BSL-18E	AAAGCGGCCGCATGCTTTTTTTTTTTTTTTTTTVN
P10EA	GGGAATTCGG
P20EA	TAATACGACTCCGAATTCCC
P22EA-ST1-R	GTCTCGTGGGCTCGGAGATGTGTATAAGAGACAGCTAATACGACTCCGAATTCCC
CA1	TGTTGAAGGCGTTTGCACATGCA
CA2	GTGCATAGACCTCATGTCTAGCA
CA-ST1-R	TCGTCGGCAGCGTCAGATGTGTATAAGAGACAGGAGGGTCAGGGTTCTGGA
CB1	GAACTGGACTTGACAGCGGAACT
CB2	AGGCAGTATCTGGAGTCATTGAG
CB-ST1-R	TCGTCGGCAGCGTCAGATGTGTATAAGAGACAGGCTCAAACACAGCGACCTC

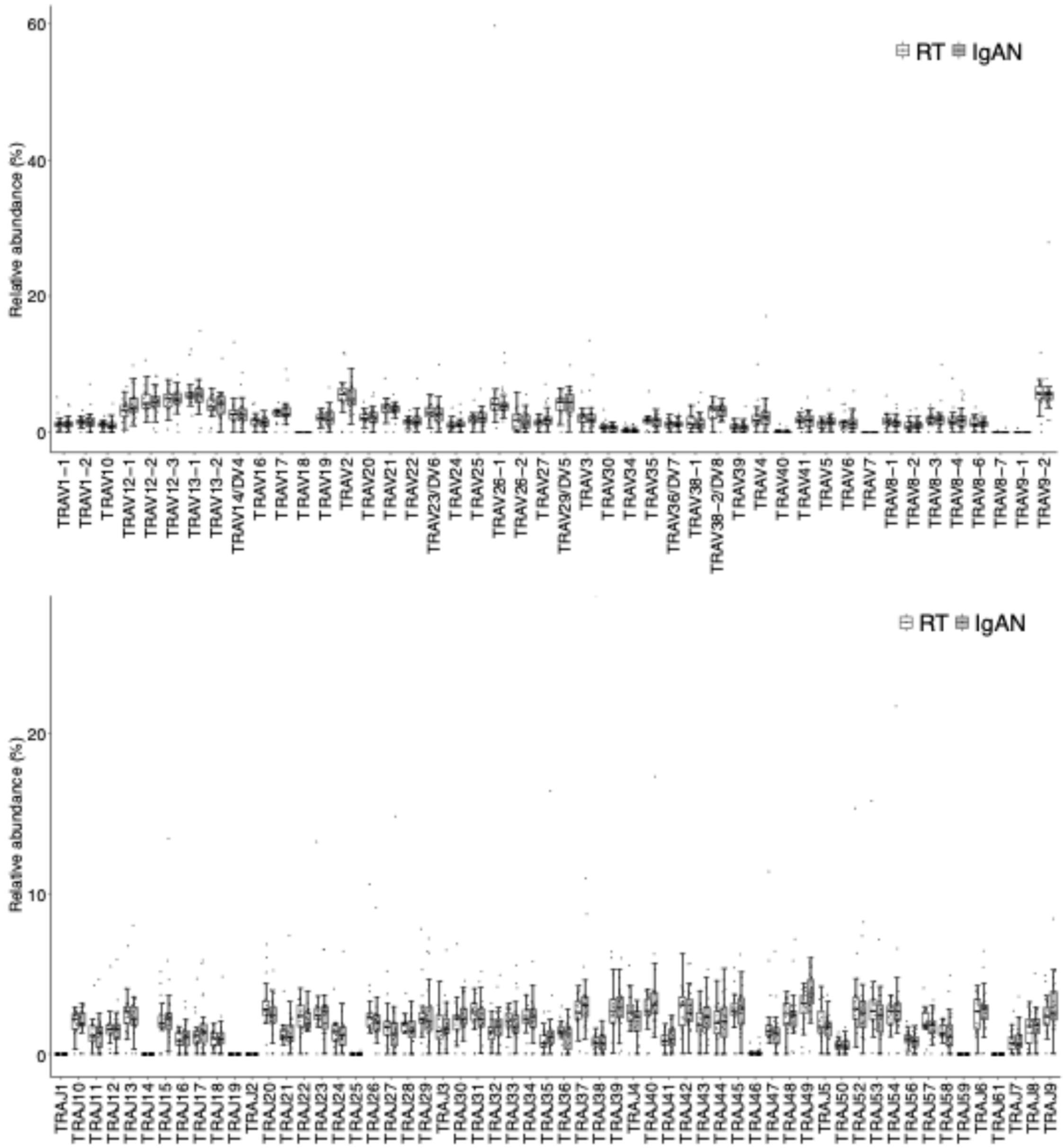

Supplemental Figure 1: Comparison of the relative abundance of TRAV and TRAJ with in-frame clones in patients with IgAN (n = 27) and patients with RT (n = 20). Group differences are assessed using the Mann–Whitney U test with false discovery rate correction (Benjamini–Hochberg method) for multiple comparisons. Data are shown as median and IQR (Mann–Whitney *U* test).

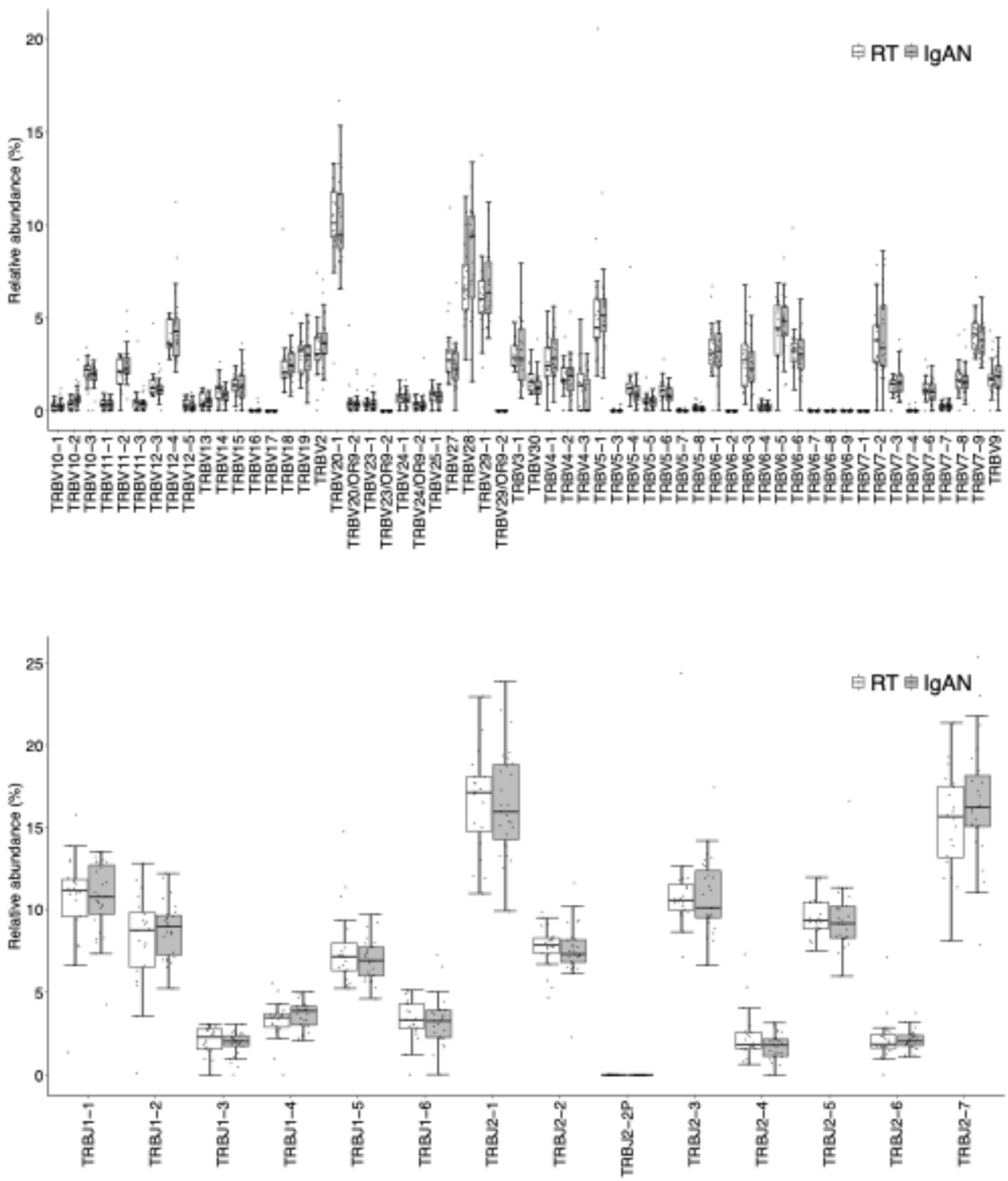

Supplemental Figure 2: Comparison of the relative abundance of TRBV and TRBJ with in-frame clones in patients with IgAN (n = 27) and patients with RT (n = 20). Group differences are assessed using the Mann–Whitney U test with false discovery rate correction (Benjamini–Hochberg method) for multiple comparisons. Data are shown as median and IQR (Mann–Whitney *U* test).

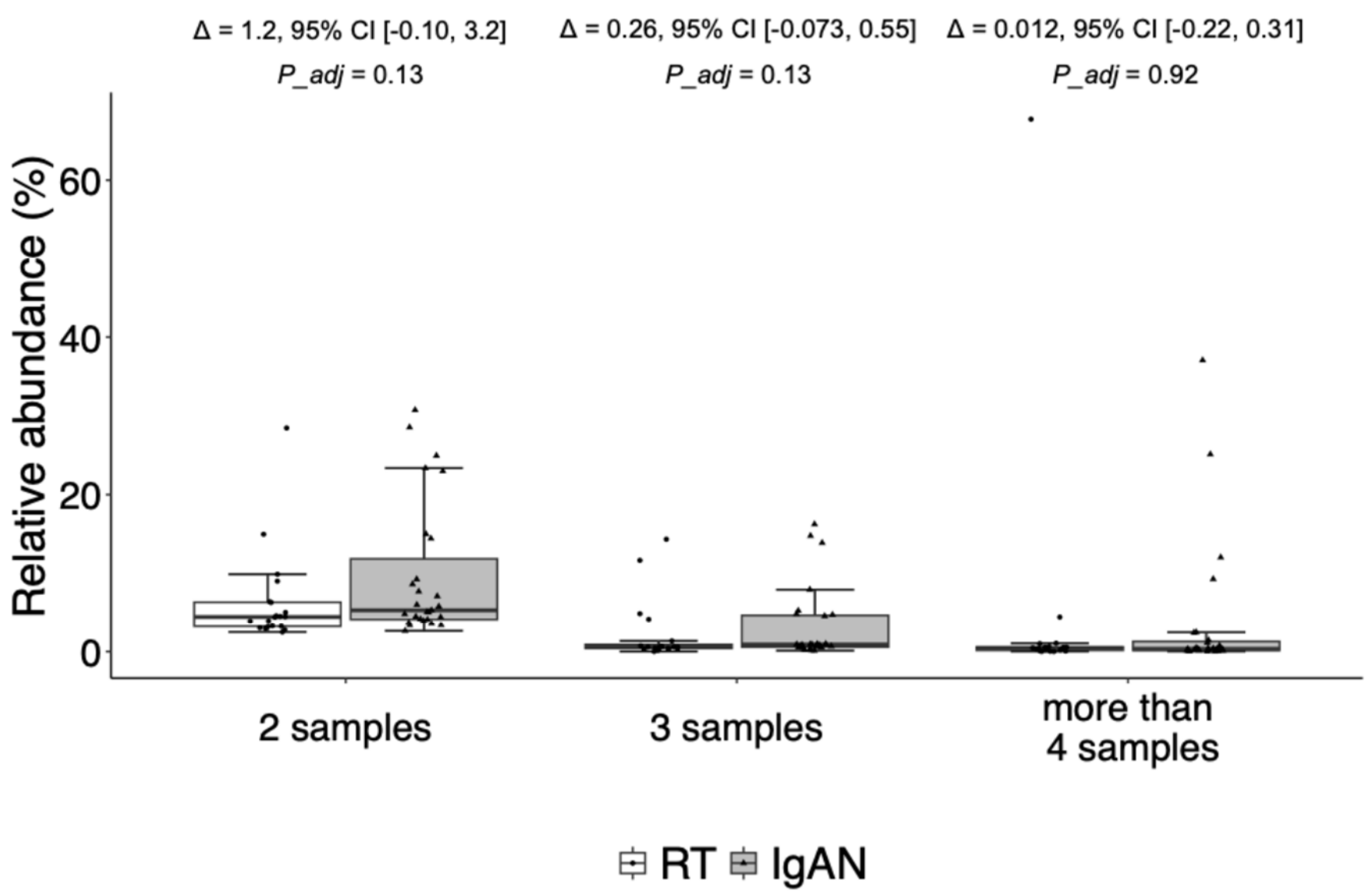

Supplemental Figure 3: Comparison of the relative abundances of public TRA repertoires shared between two, three, and more than four samples from patients with IgAN (n = 27) and patients with RT (n = 20). Group differences are assessed using the Mann–Whitney U test with false discovery rate correction (Benjamini–Hochberg method) for multiple comparisons. Reported statistics include effect sizes, 95% confidence interval (CI), and *P* values adjusted for multiple comparisons. Data are shown as median and IQR (Mann–Whitney *U* test).

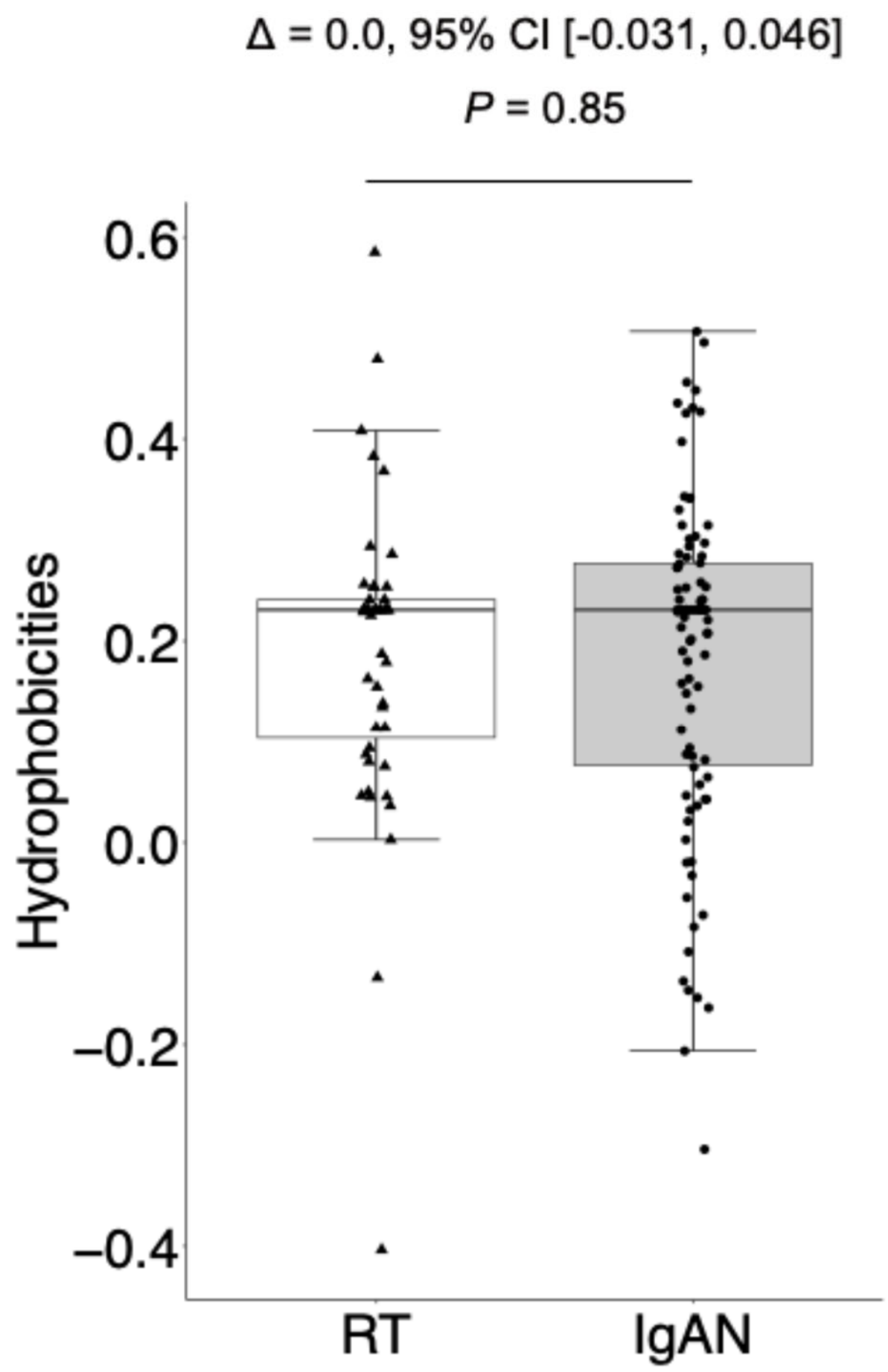

Supplemental Figure 4: Comparison of the hydrophobicity of IgAN-associated TRAs in patients with IgAN (n = 27) and patients with RT (n = 20). Group differences are assessed using the Mann–Whitney U test. Reported statistics include effect sizes, 95% confidence interval (CI), and *P* value. Data are shown as median and IQR (Mann–Whitney *U* test).

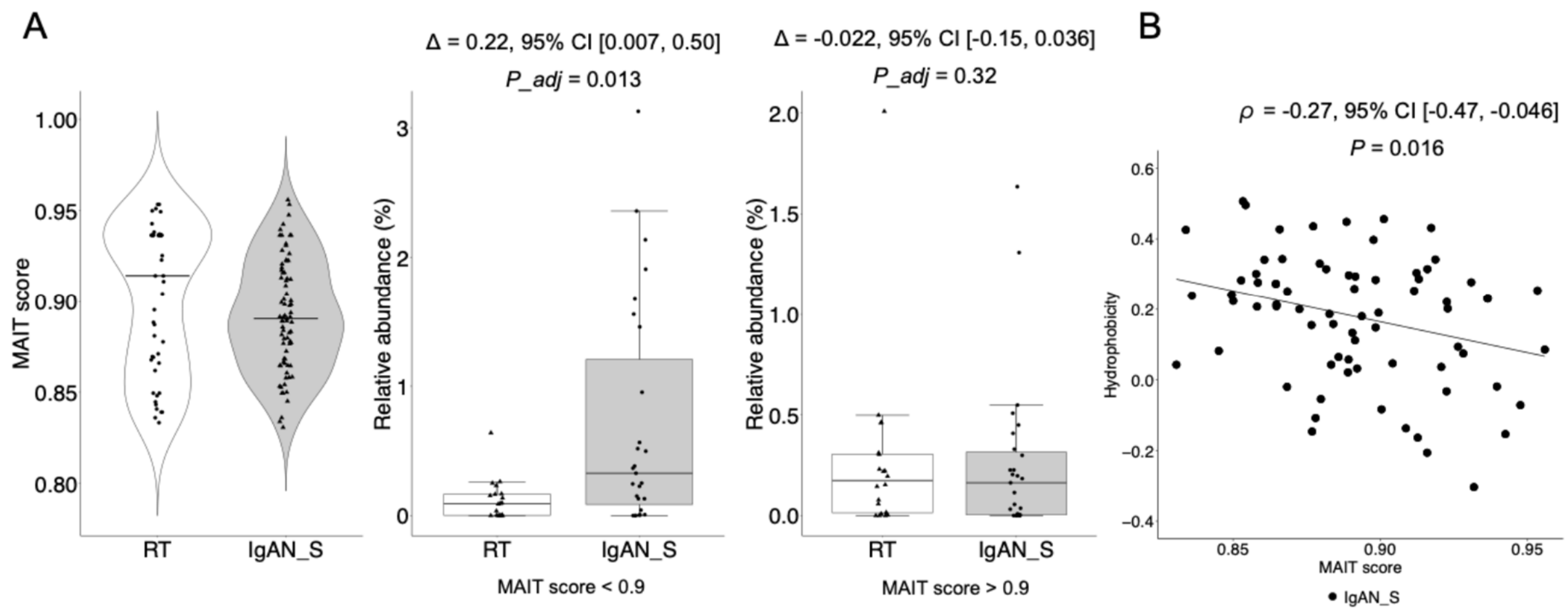

Supplemental Figure 5. Features of CDR3 amino-acid composition in clonotypes uniquely observed in patients with IgAN and comparison between patients with IgAN and patients with RT (A) Distribution of MAIT match scores for clonotypes uniquely observed in patients with IgAN (left). The relative abundance of clonotypes uniquely observed in patients with IgAN with MAIT scores < 0.9 (center) and > 0.9 (right) is compared using the Mann–Whitney U test with false discovery rate (FDR) correction (Benjamini–Hochberg) for multiple comparisons. Reported statistics include effect sizes (Δ), 95% confidence intervals (CIs), and FDR-adjusted *P* values.
(B) Correlation between MAIT match scores and hydrophobicity for clonotypes uniquely observed in patients with IgAN. Correlations are assessed using Spearman's rank correlation. Reported statistics include effect sizes (ρ), 95% CIs, and *P* values.
IgAN_S denotes TRA repertoires uniquely observed in patients with IgAN, defined by specific TRAV/TRAJ/CDR3 sequences.

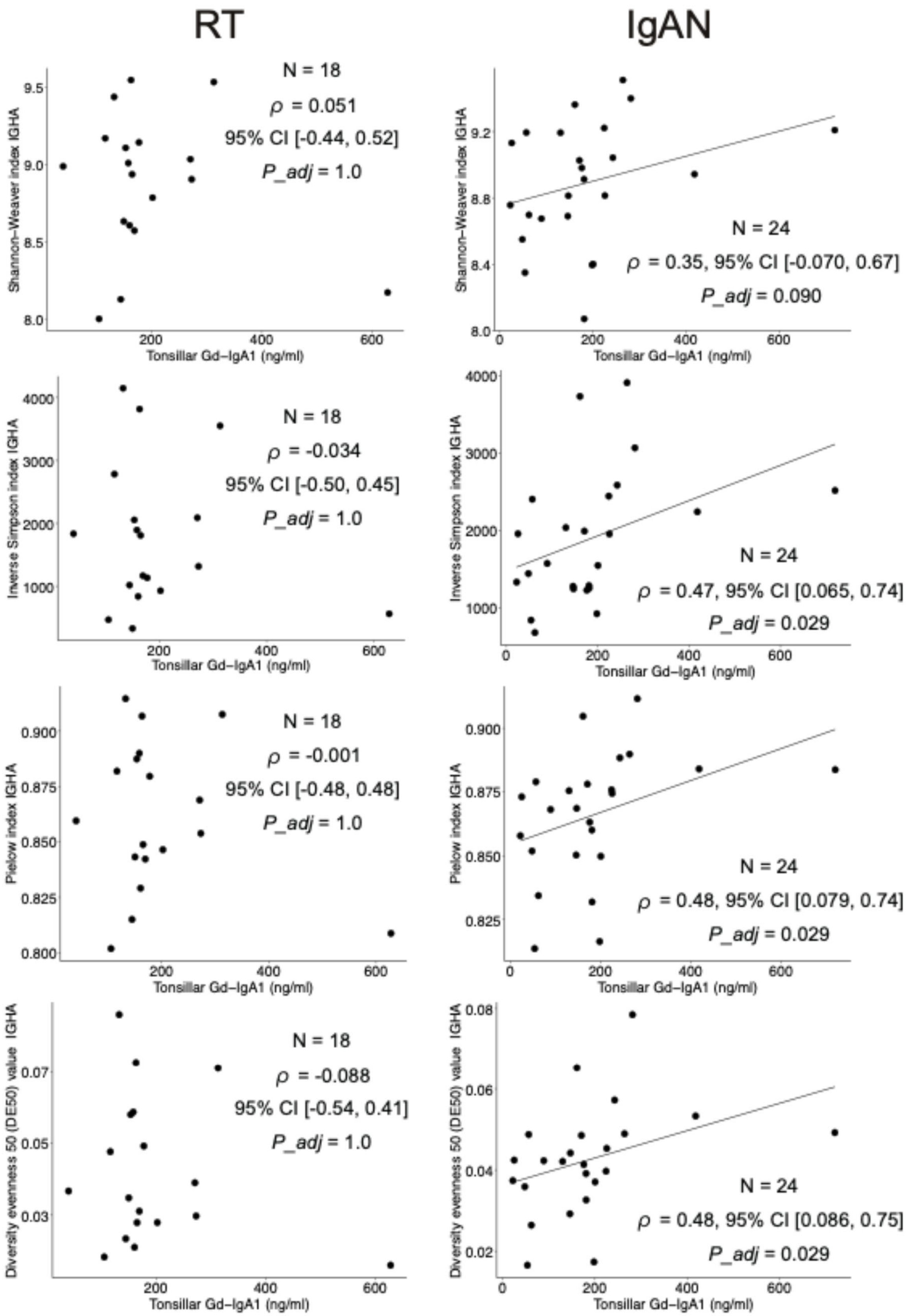
RT
IgAN
N = 18
ρ = 0.051
95% CI [-0.44, 0.52]
P_adj = 1.0
Shannon−Weaver index IGHA
Tonsillar Gd−IgA1 (ng/ml)
N = 24
ρ = 0.35, 95% CI [-0.070, 0.67]
P_adj = 0.090
Shannon−Weaver index IGHA
Tonsillar Gd−IgA1 (ng/ml)
N = 18
ρ = -0.034
95% CI [-0.50, 0.45]
P_adj = 1.0
Inverse Simpson index IGHA
Tonsillar Gd−IgA1 (ng/ml)
N = 24
ρ = 0.47, 95% CI [0.065, 0.74]
P_adj = 0.029
Inverse Simpson index IGHA
Tonsillar Gd−IgA1 (ng/ml)
N = 18
ρ = -0.001
95% CI [-0.48, 0.48]
P_adj = 1.0
Pielow index IGHA
Tonsillar Gd−IgA1 (ng/ml)
N = 24
ρ = 0.48, 95% CI [0.079, 0.74]
P_adj = 0.029
Pielow index IGHA
Tonsillar Gd−IgA1 (ng/ml)
N = 18
ρ = -0.088
95% CI [-0.54, 0.41]
P_adj = 1.0
Diversity evenness 50 (DE50) value IGHA
Tonsillar Gd−IgA1 (ng/ml)
N = 24
ρ = 0.48, 95% CI [0.086, 0.75]
P_adj = 0.029
Diversity evenness 50 (DE50) value IGHA
Tonsillar Gd−IgA1 (ng/ml)

Supplemental Figure 6: Correlations between tonsillar Gd-IgA1 levels and the diversity indices of the IGHA repertoire in tonsils of patients in both groups are statistically assessed using Spearman's correlation and linear regression analysis. *P* values are adjusted for multiple testing using false discovery rate correction (Benjamini–Hochberg method). Reported statistics include effect sizes (ρ), 95% confidence interval (CI), and *P* values adjusted for multiple comparisons.

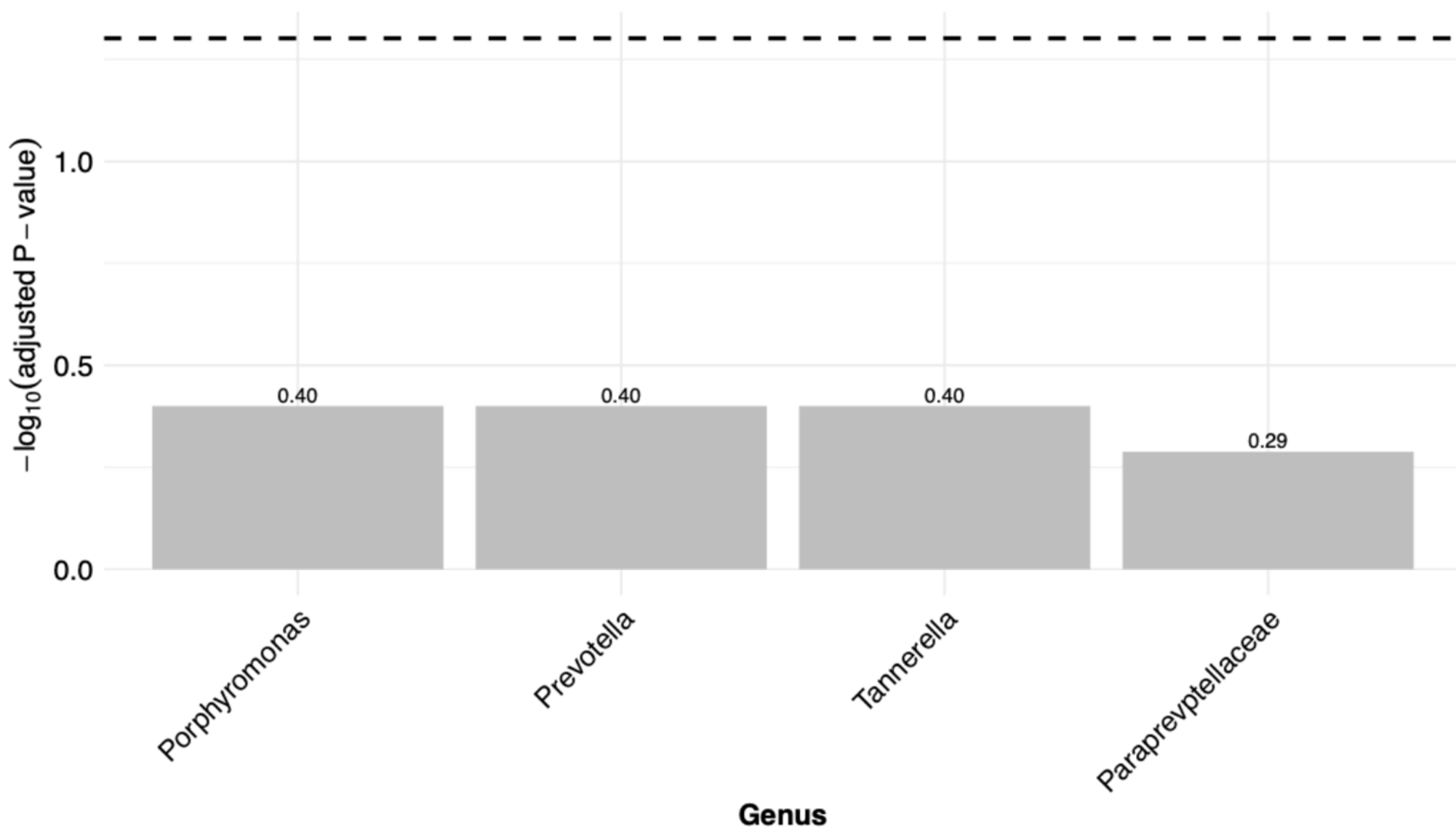

Supplemental Figure 7: Spearman's correlation analysis performed to assess the association between the relative abundance of IgAN-associated TRAs and the IgA-binding index of *Bacteroidetes* genera in the tonsils. The resulting *P* values are adjusted using false discovery rate correction (Benjamini–Hochberg method) and visualized in log-transformed form ($-\log_{10}$ (adjusted P value)), as shown. A dotted line indicates the level of significance for the adjusted *P* value.
